# Herb-drug interactions: the influence of essential oil of caraway (*Carum carvi* L.) on the pharmacokinetics of paracetamol

**DOI:** 10.1186/2050-6511-13-S1-A27

**Published:** 2012-09-17

**Authors:** Isidora Samojlik, Kornelia Ðaković-Švajcer, Biljana Božin, Momir Mikov

**Affiliations:** 1 Department of Pharmacology and Toxicology, Faculty of Medicine, University of Novi Sad, 21000 Novi Sad, Serbia; 2 Department of Pharmacy, Division of Pharmacognosy, Faculty of Medicine, University of Novi Sad, 21000 Novi Sad, Serbia

## Background

Despite the widespread use of herbal medicines, documented herb-drug interactions are sparse. Caraway (*Carum carvi* L.) is an aromatic plant from the Apiaceae family, widely used to flavor foods, as addition to fragrances, and for medical preparations. This survey examined the effects of chronic caraway essential oil pretreatment on paracetamol pharmacokinetics in male mice.

## Methods

The essential oil (EO) of caraway, prepared as emulsion for peroral use, was applied to male mice during 5 consecutive days. Paracetamol, in the dose of 200 mg/kg, was applied p.o. or i.p. 2 hours after the last EO dose. Blood samples for pharmacokinetic assay were collected from the tail vein before paracetamol intake and 10, 30, 60, 90 and 120 min thereafter. Blood concentrations of paracetamol were determined by HPLC and the pharmacokinetic parameters were calculated using the WinNonlin software.

## Results

In the control group, pharmacokinetic parameters of paracetamol after p.o. and i.p. application were rather congruent. Caraway EO pretreatment induced a statistically significant augmentation of pharmacokinetic parameters (C_max_, AUC, AUC_∞_) of i.p. applied paracetamol, speaking in favor of enhanced body exposure to the drug. However, after p.o. application of paracetamol, the pharmacokinetic data showed a significant decrease compared to control values, indicating a decrease in drug presence in the organism.

## Conclusions

The chronic intake of caraway EO influences the pharmacokinetic properties of both orally and intraperitoneally applied paracetamol. Further investigation of the exact pathway of this herb-drug interaction is needed, as well as the assessment of its real clinical significance.

